# Transformation of Sexually Transmitted Infection-Causing Serovars of *Chlamydia trachomatis* Using Blasticidin for Selection 

**DOI:** 10.1371/journal.pone.0080534

**Published:** 2013-11-26

**Authors:** Honglei Ding, Siqi Gong, Yingxin Tian, Zhangsheng Yang, Robert Brunham, Guangming Zhong

**Affiliations:** 1 Department of Microbiology and Immunology, University of Texas Health Science Center at San Antonio, San Antonio, Texas, United States of America; 2 BC Centre for Disease Control, University of British Columbia, Vancouver, British Columbia, Canada; Oregon State University, United States of America

## Abstract

Plasmid-free *Chlamydia trachomatis* serovar L2 organisms have been transformed with chlamydial plasmid-based shuttle vectors pGFP::SW2 and pBRCT using β-lactamase as a selectable marker. However, the recommendation of amoxicillin, a β-lactam antibiotics, as one of the choices for treating pregnant women with cervicitis due to *C. trachomatis* infection has made the existing shuttle vectors unsuitable for transforming sexually transmitted infection (STI)-causing serovars of *C. trachomatis*. Thus, in the current study, we modified the pGFP::SW2 plasmid by fusing a blasticidin S deaminase gene to the GFP gene to establish blasticidin resistance as a selectable marker and replacing the β-lactamase gene with the *Sh ble* gene to eliminate the penicillin resistance. The new vector termed pGFPBSD/Z::SW2 was used for transforming plasmid-free *C. trachomatis* serovar D organisms. Using blasticidin for selection, stable transformants were obtained. The GFP-BSD fusion protein was detected in cultures infected with the pGFPBSD/Z::SW2-trasnformed serovar D organisms. The transformation restored the plasmid property to the plasmid-free serovar D organisms. Thus, we have successfully modified the pGFP::SW2 transformation system for studying the biology and pathogenesis of other STI-causing serovars of *C. trachomatis*.

## Introduction

The species *Chlamydia trachomatis* consist of many serovars with 3 distinct types of tissue tropism. Serovars A to C mainly infect ocular epithelial cells [1] while serovars L1 to 3 mainly invade genital and rectal epithelial tissues, which can result in disseminated infections [2]. Serovars D to K target genital tract epithelial cells for infection and are the leading cause of sexually transmitted bacterial infection. Infection with serovars D to K in the female lower genital tract can cause cervicitis although frequently accompanying no apparent clinic symptoms. However, if untreated, the lower genital tract infection can lead to ascending infection termed pelvic inflammatory disease (PID), which in turn can be symptomatic or subclinical. Some PID patients develop late complications such as ectopic pregnancy and tubal factor infertility [3]. Thus, timely treatment of cervical infection is the key to prevent complications including preterm delivery among pregnant women [4]. Amoxicillin, a β-lactam antibiotics, has been recommended as one of the choices for treating pregnant women with Chlamydia (http://www.cdc.gov/std/treatment/2010/chlamydial-infections.htm#chlampreg).

All *C. trachomatis* serovars regardless of their tissue tropism share an identical intracellular growth cycle. It has been proposed that intracellular growth of Chlamydia triggers inflammatory responses that contribute significantly to disease pathogenesis [5-8]. *C. trachomatis* intracellular infection starts with an infectious elementary body (EB) entering an epithelial cell via endocytosis. The intravacuolar EB rapidly differentiates into a reticulate body (RB) that is no longer infectious but is metabolically active which undergoes replication. Progeny RBs eventually differentiate back to EBs prior to exiting infected cells and spreading to new cells. Intracellular Chlamydia restrict themselves within the cytoplasm to membrane-limited vacuoles that are also called inclusions. It has been difficult to identify chlamydial virulence molecules and to determine how these molecules induce pathology-causing inflammation due to lack of convenient genetic tools. 

 Recently, a chlamydial plasmid shuttle vector-based transformation system has been developed [9-13]. This technology has led to the determination of the molecular basis of plasmid-dependent pathogenesis [10-12] and can be used to introduce genes into Chlamydia [11]. However, the shuttle vectors used in these experiments carry a β-lactamase gene, which if transferred to wild type strains of *C. trachomatis* would limit usefulness of beta lactam antibiotics for this class of organism. The goal of the current study is to modify one of the existing shuttle vectors, pGFP::SW2, for transforming the STI-causing serovars. We fused a blasticidin S deaminase gene to the GFP gene in pGFP::SW2 to establish blasticidin resistance as a primary selectable marker and replaced the β-lactamase gene in pGFP::SW2 with the *Sh ble* gene to eliminate the penicillin resistance. The new vector designated as pGFPBSD/Z::SW2 was used for transforming plasmid-free *C. trachomatis* serovar D with blasticidin for selection. The GFP-BSD fusion protein was detected in pGFPBSD/Z::SW2-transformed serovar D. The transformation restored the plasmid property to a plasmid-free serovar D strain of *C. trachomatis*. Thus, we successfully constructed a modified vector with β-lactamase-free selectable markers for future studies of the biology and pathogenesis of STI-causing serovars of *Chlamydia trachomatis*. Such knowledge is essential for the understanding of chlamydial pathogenic mechanisms and the development of chlamydial vaccines.

## Materials and Methods

### 1. Cell culture and chlamydial infection

HeLa (human cervical epithelial carcinoma cells, ATCC cat# CCL2), McCoy cells (mouse fibroblast cell, CRL-1696), L929 cells (mouse fibroblast cell, NCTC clone 929, CCL-1) and *C. trachomatis* serovar D (UW-3/Cx, VR-885) were purchased from ATCC. The chlamydial organisms were propagated, purified, aliquoted and stored as described previously [14,15]. For infection, cells grown in 24 well plates with coverslips or 6 well plate containing DMEM (Sigma, St. Louis, MO) with 10% fetal bovine serum (FBS, Gemini Bio-products, West Sacramento, CA) at 37°C in an incubator supplied with 5% CO2 were inoculated with chlamydia as described previously [16]. 

### 2. Constructing recombinant plasmids with a β-lactamase-free selectable marker

Primer pairs listed in Table S1 were used to amplify a blasticidin S deaminase gene (BSD) from the vector pLenti6.3_V5-TOPO [Life Technologies, Grand Island, NY 14072; ref: [17]], a *Sh ble* gene (ZeoR) from pFuse-hLgG1-FC2 vector (INVIVOGEN, San Diego, CA92121) and DNA fragments lacking both the chloramphenicol acetyltransferase (CAT) and β-lactamase (bla) genes using pGFP::SW2 [kindly provided by Dr. Ian Clark, ref: [9]] as template. The *Sh ble* gene is initially from *Streptoalloteichus hindustanus* and encodes a protein that binds to zeocin, inhibiting the DNA cleavage activity of zeocin, thus, *Sh ble* is also referred to as ZeoR. The PCR reaction conditions used were: initial denaturation at 95°C for 3 min followed by 35 cycles of amplification at 95°C 15s, 55°C for 30s and 68°C for 1 min per kb and a final extension at 68°C for 10min. PCR products were purified using the GeneJET PCR Purification Kit (Fisher Scientific, Pittsburgh, PA) and digested with the FastDigest DpnI (Fisher Scientific, Pittsburgh, PA) to remove template DNA. An in-fusion HD cloning kit (Clontech Laboratories Inc, Mountain View, CA) was used to fuse the PCR products according to the manufacturer’s instruction. The fusion products were transformed into Stellar Competent Cells (included in Clontech In-Fusion HD cloning Kit) and the transformants were selected for zeocin (INVIVOGEN) on a LB agar plate. Bacterial colonies were screened for positive green fluorescence by visualization under fluorescence microscopy and replacement of CAT and bla with BSD and ZeoR respectively as detected by PCR (the screening PCR primers are also listed in Table S1). Plasmids were extracted from the colonies with the desired screening results and the purified plasmids were transformed into *E. coli* K12 ER2925 (Dam^-^ Dcm^-^ strain, New England Biolabs, Ipswich, MA) for amplification. The final plasmid after DNA sequencing validation was designated as pGFPBSAD/Z::SW2 and used for chlamydial transformation. The sequence of the plasmid pGFPBSD/Z::SW2 has been deposited in GenBank.

### 3. Plaque-forming assay and generation of plasmid-free *C. trachomatis* *serovar* D strains

The plaque assay was carried out as described previously [18] with some modifications. Briefly, the survival *Chlamydia trachomatis* serovar D harvested from novobiocin-treated cultures were serially diluted and inoculated onto confluent monolayers of McCoy cells in 6-well plates. The infection was facilitated by centrifugation at 1,500 rpm for 1 h at 37°C. The inoculum was replaced with DMEM containing 1 μg/ml of cycloheximide and 10% FBS. Four hours later, the medium was replaced with agarose overlay medium (1xEMEM medium, 3.5 g/L glucose, 2 mM L-glutamine, 1mM sodium pyruvate, 10% FBS, 10 μg/ml gentamicin, 2μg/ml cycloheximide, and a final concentration of 0.55% of agarose) followed by dispensing 4ml of pre-warmed DMEM supplemented with 10% FBS on top of the agarose layer. The cultures were then incubated for 5 to 7 days to allow plaques to form. Individual plaques (well separated) were picked up into 100 μl of SPG buffer for making lysates by vortexing with glass beads. Portions of the lysates were used for infecting HeLa monolyaers grown in 96 well microplates for screening for lack of Pgp3 expression using an immunofluorescence assay as described below. Five independent clones designated as CTD1 to 5 were identified as Pgp3-negative. These Pgp3-negative clones were further validated for lack of glycogen accumulation and plasmid genes. To ensure monoclonality, each of the 5 clones was replaqued twice and the final clones were purified for further experiments, including transformation. 

### 4. Transforming plasmid-free *C. trachomatis* *serovar* D

The transformation was performed using transformation protocol developed previously [11] with modifications. Briefly, 10μl plasmid-free serovar D (clone CTD1.31, 1 x 10^7^ IFU) and 10μl plasmid DNA (pGFPBSD/Z::SW2, ~7μg) were mixed in a total volume of 200μl CaCl_2_ buffer for 45 minutes at room temperature. Freshly trypsinized L929 cells (6 x 10^6^ cells) re-suspended in 200μl CaCl_2_ buffer were added to the EB/plasmid mixture and incubated for a further 20 min at room temperature with occasional mixing. Each 70μl of the final mixture was plated to a single well of a six-well plate together with 1.5 ml of pre-warmed DMEM + 10% FBS. The cells were allowed to adhere to the culture plate by incubating at 37°C in 5% CO_2_ for 24h without cycloheximide (Sigma-Aldrich, St. Luis, MO) or blasticidin (INVIVOGEN). The cultures were replenished with fresh DMEM-10% FBS containing cycloheximide (2 µg/ml) and blasticidin (5 µg/ml) and incubated an additional 24h. Inclusions positive for green fluorescence were identified under an inverted fluorescence microscope and marked at the bottom of the plate with a marker pen. Cells from the marked area were scraped off (without detaching the rest of the cells in the plate) and harvested into 200 µl SPG buffer in a 1.5ml Eppendorf vial that contains a grass bead. After a brief vortexing, the suspension was passed to a fresh monolayer of HeLa cells in a 24-well plate. After incubation at 37°C for 2h, the initial inoculum was removed and the well was supplied with 1ml DMEM-10% FBS containing cycloheximide (2 µg/ml) and blasticidin (20μg/ml). Blasticidin at 20 µg/ml inhibited the growth of green fluorescence-negative CTD1 inclusions without obvious toxicity to host cells and did not inhibit the expansion of CTD1 inclusions positive for green fluorescence. The culture was continued at 37°C. Green fluorescence-positive inclusions were identified again under a fluorescence microscope 48h after incubation. The green fluorescence-positive inclusions, defined as generation two, were harvested and passed as described above for additional 3-4 generations before they were subjected to plaque-purification as described previously [19]. The chlamydial stable transformants were designated as CTD1-pGFPBSD. 

### 5. Live cell culture microscopy and indirect immunofluorescence assay

An IX-81 inverted fluorescence microscope (Olympus, Center Valley, PA) was used to visualize live cells without or with infection by serovar D wild type, plasmid-free CTD1.31 or CTD1-pGFPBSD transformant organisms. Simple PCI imaging software (Olympus) was used to acquire both bright field and green fluorescence images and Adobe Photoshop (Adobe, San Jose, CA) was used to do post-acquisition processing of the images.

For immunofluorescence images, HeLa cells with or without chlamydial infection were fixed at 48 hours post infection with 2% paraformaldehyde dissolved in PBS for 30 min at room temperature, followed by permeabilization with 2% saponin (Sigma, St. Louis, MO) for an additional 30min. After blocking, the cell samples were subjected to antibody and chemical staining. Hoechst 33258 (blue, Sigma-Aldrich) was used to visualize DNA (blue). A rabbit anti-chlamydial organism antibody (R1L2, ref [20]: plus a goat anti-rabbit IgG secondary antibody conjugated with Alexa Fluor 488 (green; Jackson ImmunoResearch, West Grove, PA) was used to visualize chlamydia. The mouse anti-Pgp3 or GlgA (glycogen synthase A) antibodies (raised with GST fusion proteins, unpublished data) plus a goat anti-mouse IgG conjugated with Cy3 (red; Jackson ImmunoResearch) were used to visualize Pgp3 and GlgA respectively. The immunofluorescence images were acquired using an Olympus AX-70 fluorescence microscope equipped with multiple filter sets and Simple PCI imaging software as described previously [21]. 

### 6. Iodine staining

HeLa cells with or without chlamydial infection were fixed with ice-cold methanol for 10 min. The cell samples were stained with 5% iodine stain (5% potassium iodide and 5% iodine in 50% ethanol) for 40 min. The coverslips were mounted in 50% glycerol containing 5% potassium iodide and 5% iodine. The images were acquired by using Olympus CH-30 microscope equipped with Canon EOS Rebel T3i Digital SLR Camera. The images were processed using Adobe Photoshop.

### 7. PCR

Chlamydia were lyzed with 0.1% SDS and lysates were used as PCR templates after dilution at 1:1000. The 8 plasmid-encoded and one genomic gene-specific primers used in the current study are listed in Table S1. The PCR conditions were as followings: initial denaturation @ 94°C for 5min followed by 30 cycles at 94°C for 30s, 50°C for 45s and 72°C for 2min. One third of the final PCR products amplified from each template were run on agarose gels. 

## Results

### 1. Construction of a shuttle vector with a β-lactamase-free selectable marker for transforming STI-causing serovars of *C. trachomatis*

**Figure 1:**
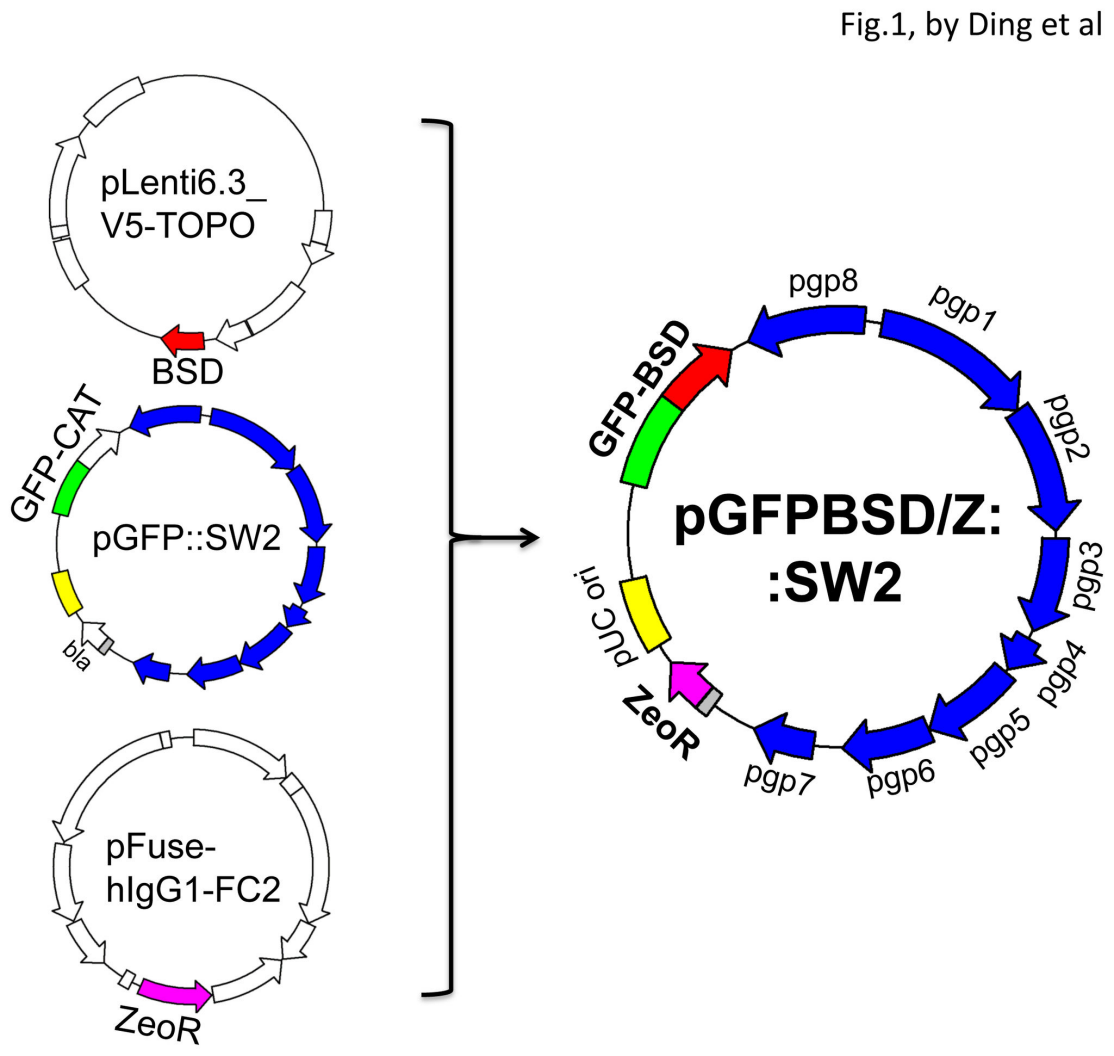
Construction of a shuttle vector with a β-lactamase-free selectable marker. A blasticidin S deaminase gene (BSD) from the vector pLenti6.3 was used to replace the Chloramphenicol acetyltransferase gene (CAT) in the pGFP::SW2 vector to produce a GFP-BSD fusion construct. At the same time, the Sh *ble* gene from the pFuse vector was used to replace the β-lactamase gene (bla) in the pGFP::SW2 to eliminate penicillin resistance. The resultant plasmid vector termed pGFPBSD/Z::SW2 is thus free of β-lactamase and CAT but with a new selectable marker.

To develop a shuttle vector that can be used for transforming STI-causing serovars of *C. trachomatis*, we modified the pGFP::SW2 plasmid by first using a blasticidin S deaminase gene (BSD) from the vector pLenti6.3 [17] to replace the chloramphenicol acetyltransferase gene (CAT) as a fusion partner of GFP (Figure 1). This genetic replacement was designed for the vector to code for a GFP-BSD fusion protein since the fusion is known to be stable and to maintain BSD’s deaminase function [22]. In this way, the plasmid can be selected for by using blasticidin. Furthermore, the ShSh ble gene [23] was cloned to replace the β-lactamase gene in the pGFP::SW2 for eliminating penicillin resistance. Thus, the resultant plasmid vector termed pGFPBSD/Z::SW2 is free of β-lactamase but with a new selective marker. 

**Figure 2:**
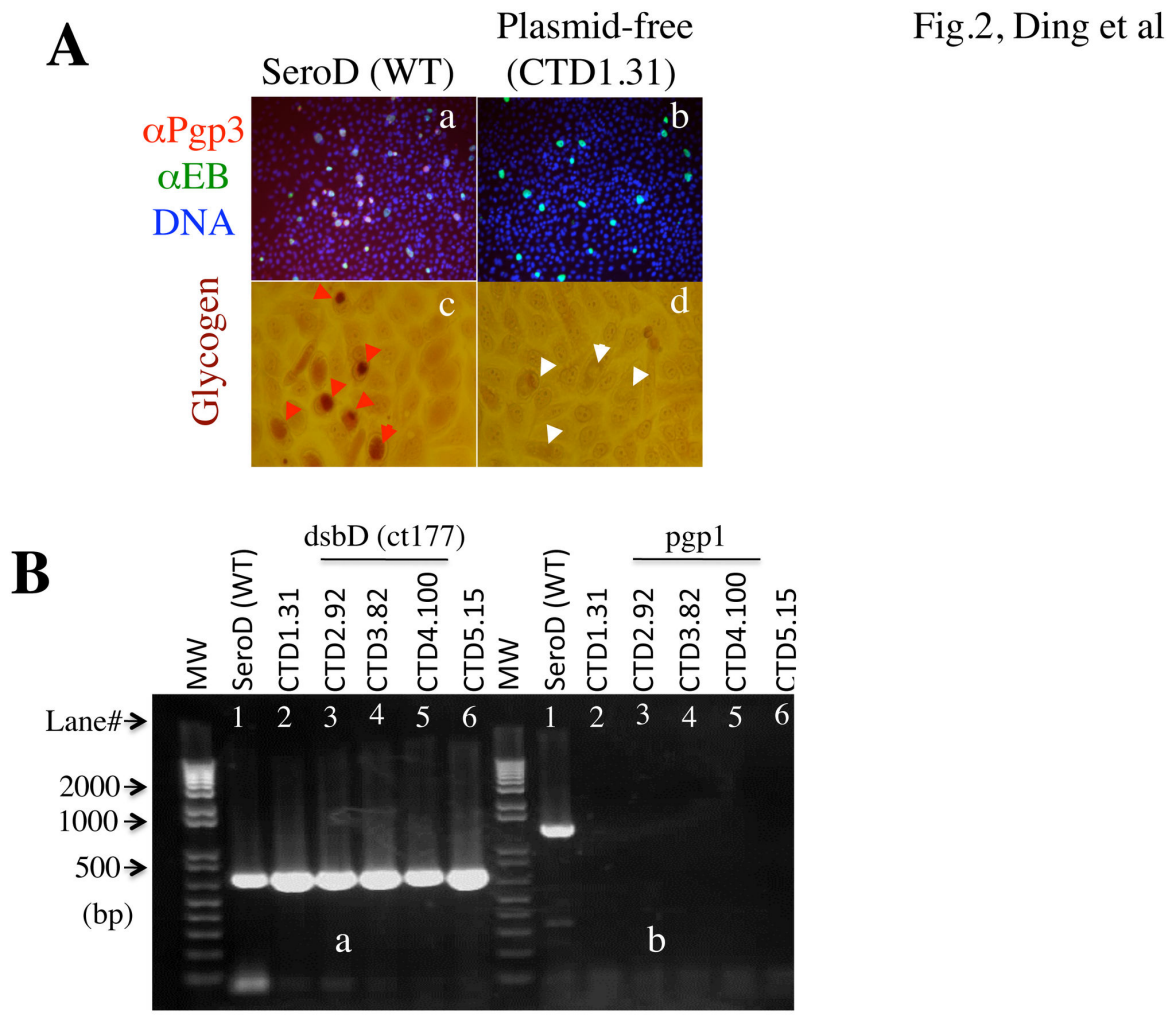
Identification of plasmid-free *C. trachomatis* serovar D. (A) A novobiocin-based selection approach was used to deplete plasmid from *C. trachomatis* serovar D organisms [SeroD (WT), panel a]. After plaque purification, 5 independent clones (clone CTD1.31 shown in panel b; the remaining 4 clones CTD2.92, CTD3.82, CTD4.100 & CTD5.15 were not shown) were identified as lack of Pgp3 protein expression. Pgp3 was labeled red, chlamydial organisms green while host nuclei blue. The 5 Pgp3-free clones also lacked glycogen accumulation in the inclusions when parallel cultures were labeled with iodine. Inclusions with positive glycogen accumulation were indicated with read arrowheads (c) while those without marked with white arrowheads (d). (B) The 5 Pgp3-free clones (lanes# 2 to 6) along with the wild type serovar D (lane#1) were further subjected to PCR detection of plasmid genes (only pgp1 gene detection result was shown in panel b). Note that although the genome-encoded gene dsbD (open reading frame ct177) was detected in both the wild type serovar D (lane#1 in all panels) and all 5 clones (panel a), no pgp1 was detected in any of the 5 Pgp3-free clones (lanes#2 to 6 in panels b).

The next step is to generate plasmid-free *C. trachomatis* serovar D as the recipient for the above β-lactamase-free shuttle plasmid (Figure 2). A novobiocin-based selection approach was used to deplete plasmid from *C. trachomatis* serovar D organisms [19,24,25]. A total of 5 independent clones (designated as CTD1.31, CTD2.92, CTD3.82, CTD4.100 & CTD5.15) were plaque-purified and identified as lacking of Pgp3 protein expression 48h after infection in HeLa cell culture. These Pgp3-free clones also lacked glycogen accumulation and 8 plasmid genes although the genome-encoded gene dsbD (open reading frame ct177) was detectable. Thus, all 5 Pgp3-free clones lacked the cryptic plasmid. 

**Figure 3:**
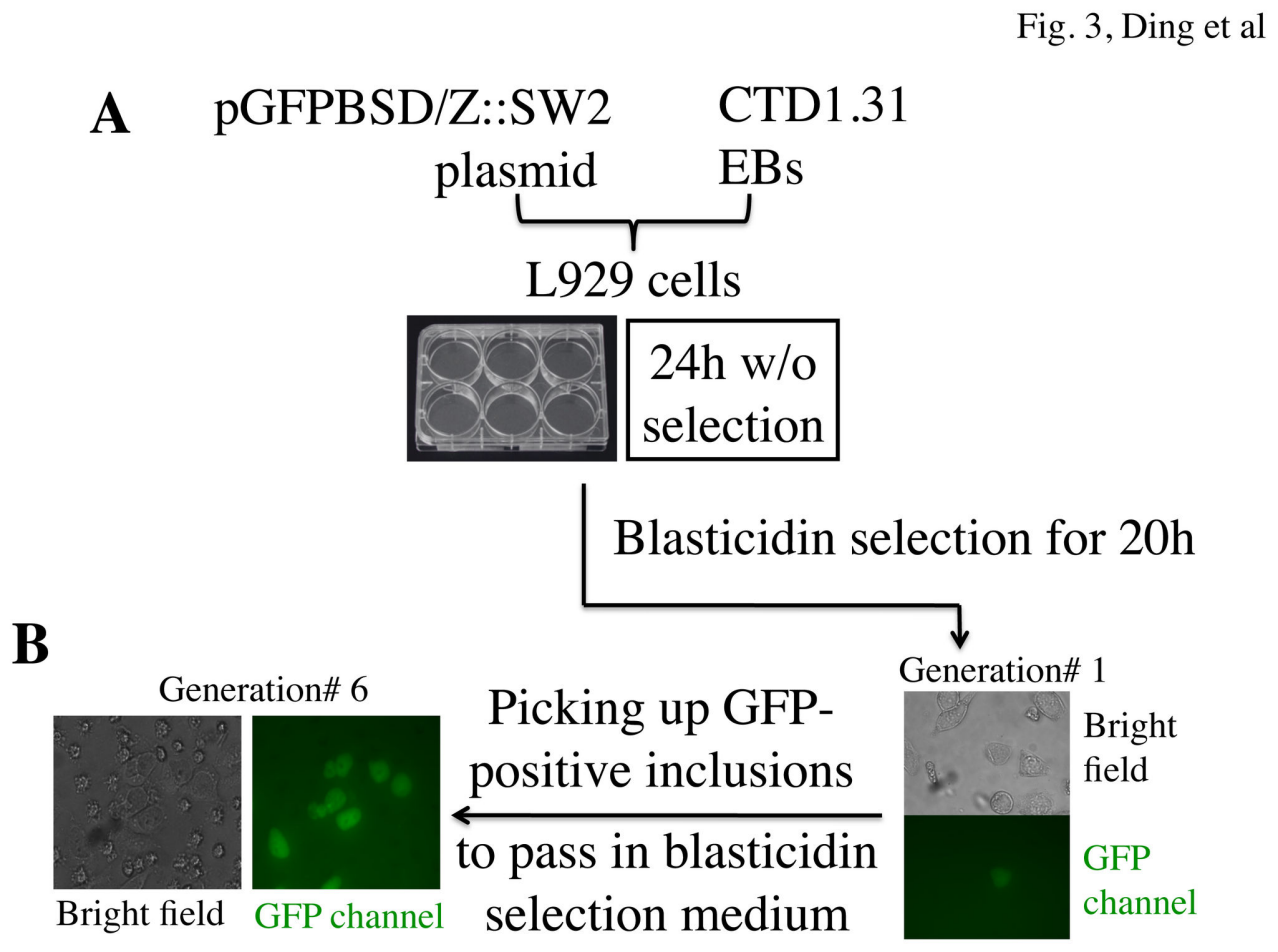
Transformation of *C. trachomatis* serovar D with the newly modified plasmid pGFPBSD/Z::SW2. (A) As described in the materials and method section, the newly modified pGFPBSD/Z::SW2 plasmid was used to transform the plasmid-free serovar D clone CTD1.31 EBs in L929 culture. The culture was incubated for 24h without blasticidin, then with blasticidin for another 20h. (B) GFP positive inclusions were picked up and passed to new cell cultures in the presence of blasticidin selection. Under passage for 6 rounds (generation#6), most inclusions became GFP positive.

Finally, the newly modified pGFPBSD/Z::SW2 plasmid was transformed to a plasmid-free serovar D clone CTD1.31 in L929 culture. As shown in Figure 3, a procedure similar to what was described previously [11] was followed. The initial culture was incubated for 24h without blasticidin, then with blasticidin for another 20h. We tried the transformation procedures 3 times. Although each transformation varied in terms of the number of GFP-positive inclusions observed in each generation, stable transformants were always obtained. If GFP positive inclusions were visible from the initially transformed culture (generation 1) under an inverted fluorescence microscope, the inclusions were picked up and passed to new cell cultures in the presence of blasticidin selection. If there was no GFP-positive inclusion in the generation 1, the cultures were blindly passed with blasticidin selection until GFP-positive inclusions were visible. In most cases, after 6 rounds of passage most inclusions became GFP positive. We used a plaque assay [19] to purify a single clone (termed CTD1-pGFPBSD) for further analyses.

### 2. Characterization of the stable transformant CTD1-pGFPBSD

**Figure 4:**
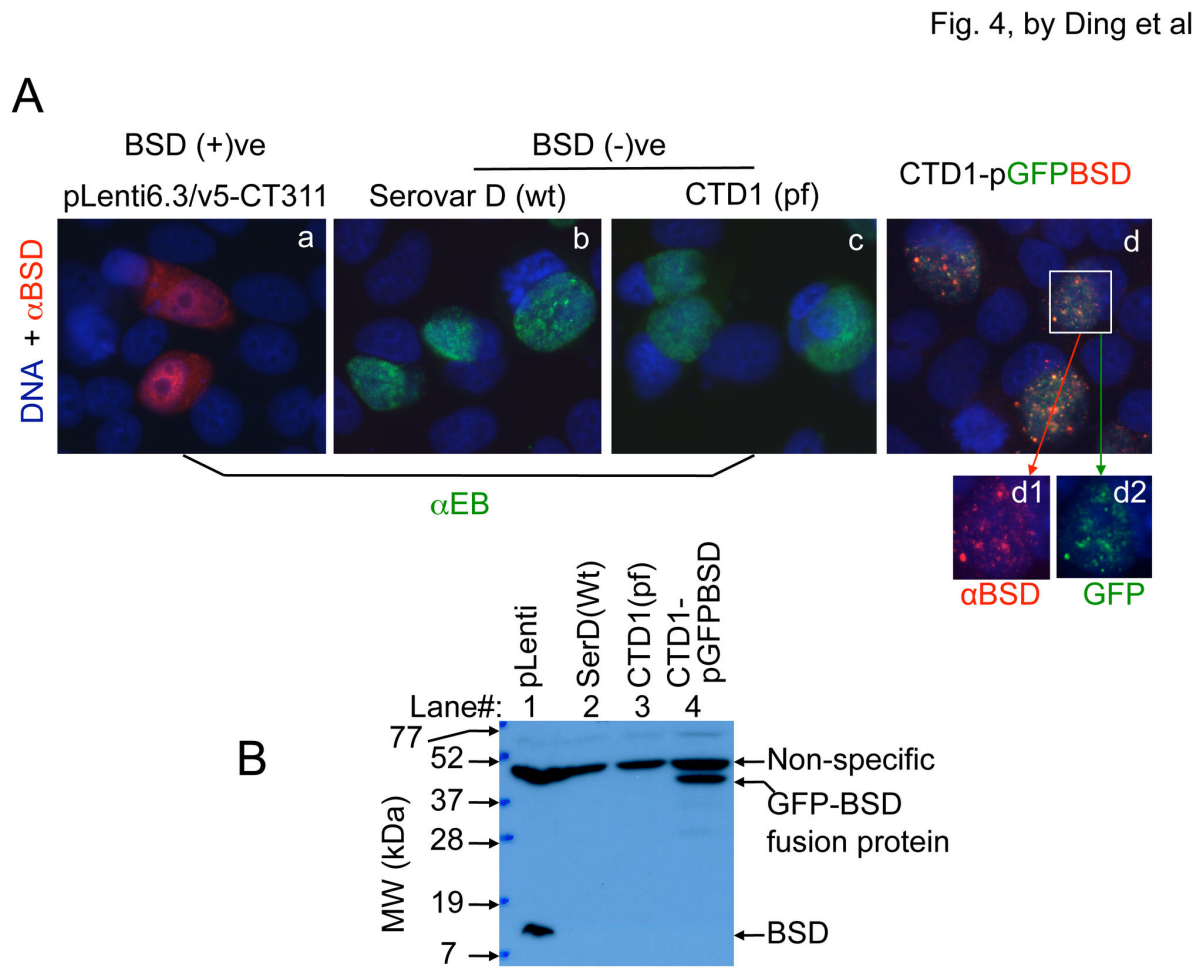
Detection of GFP-BSD fusion proteins in CTD1-pGFPBSD-infected cultures. (A) HeLa cells were either transfected with a pLenti6.3/v5-CT311 plasmid [as positive control for BSD expression, BSD (+)ve, panel a] or infected with *C. trachomatis* serovar D wild type (wt, b), the plasmid-free (pf) CTD1.31 (c) or the CTD1 transformant (CTD1-pGFPBSD, d). Both the wt and pf serovar D-infected cultures were used as a negative control for BSD expression [BSD (-)ve, a & c]. All culture samples were labeled with an anti-BSD antibody (red) and DNA dye (blue) while the pLenti6.3-transfected and wt and pf serovar D-infected cultures were further stained with a rabbit anti-EB antibody (green, a-c) and the transformant-infected culture displayed GFP green color (d). Note that both BSD and GFP were detected only in the inclusions of the CTD1-pGFPBSD transformant-infected cultures (d1 & d2). (B) The above cultures were also analyzed using a Western blot assay for the presence of GFP-BSD fusion proteins. Free BSD was detected in pLenti-transfected cultures (lane#1) while the GFP-BSD fusion protein in the transformant-infected culture (lane#4).

The stable transformant CTD1-pGFPBSD was monitored for expression of the GFP-BSD fusion protein (Figure 4). HeLa cells transfected with a pLenti6.3/v5-CT311 plasmid coding for free BSD [17] was used as positive control for BSD expression while HeLa cells infected with *C. trachomatis* serovar D wild type or plasmid-free organisms were used as negative controls for BSD expression. The anti-BSD antibody detected strong signals in both the pLenti6.3-transfected and CTD1-pGFPBSD-infected cultures, indicating that BSD was expressed in both. The parallel cultures were also analyzed using a Western blot assay for the presence of GFP-BSD fusion protein. As expected, free BSD was detected in the pLenti6.3-transfected cultures while the GFP-BSD fusion protein in the transformant-infected culture. These detection results together demonstrated that GFP-BSD fusion was expressed by the transformant CTD1-pGFPBSD. 

**Figure 5:**
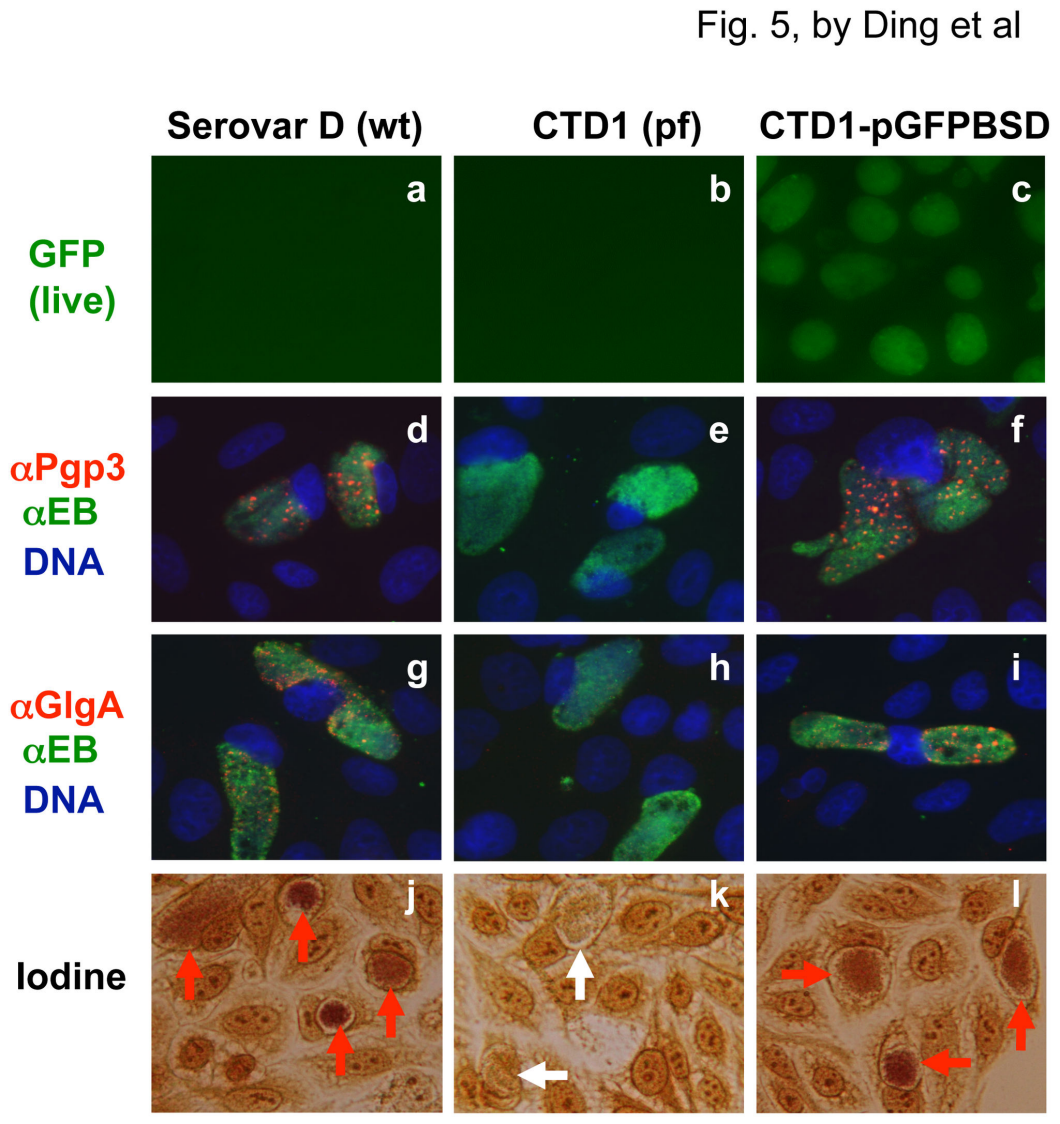
Restoration of plasmid property in CTD1-pGFPBSD transformant. HeLa cells infected with *C. trachomatis* serovar D wild type (wt, panels a, d, g & j), plasmid-free (pf, b, e, h & k) or transformant (c, f, I & l) were either observed directly for GFP (green, a-c) or subjected to immunolabeling for Pgp3 (red, d-f) or GlgA (red, g-i) or iodine staining for glycogen accumulation. Glycogen positive inclusions were marked with read arrows while glycogen negative ones with white arrows (panels j-l). The anti-Pgp3 or anti-GlgA labeled samples were co-stained with anti-EB antibody (green) and DNA dye (blue). Pgp3 and GlgA as well as glycogen accumulation were detected in cultures infected with wild type or transformant but not plasmid-free serovar D.

We further characterized the plasmid property of the CTD1-pGFPBSD transformant by detecting Pgp3 (encoded by the plasmid) and GlgA (encoded in the genome but regulated by the plasmid) protein expression as well as glycogen accumulation (Figure 5). The Pgp3 and GlgA proteins were detected in the cultures infected with either wild type *C. trachomatis* serovar D or CTD1-pGFPBSD transformant but not the plasmid-free CTD1.31 organisms. Glycogen accumulation displayed a similar distribution pattern. Thus, the transformation of the shuttle vector pGFPBSD/Z::SW2 into the plasmid-free CTD1.31 fully restored the assessed plasmid properties of the transformant. 

## Discussion

The successful transformation of *C. trachomatis* has provided new opportunities for studying chlamydial pathogenesis and developing vaccines. However, the current transformation system is based on chlamydial plasmid shuttle vectors with β-lactamase as a selection marker [9-13]. Since amoxicillin, a β-lactam antibiotic is currently recommended for treating pregnant women with *C. trachomatis* infection, the current shuttle vectors should not be used for transforming STI-causing serovars of *C. trachomatis*. A newly modified plasmid was constructed and designated as pGFPBSD/Z::SW2 with the following improvements: A blasticidin S deaminase gene was used to replace the CAT gene and fuse to the GFP gene in pGFP::SW2, which led to the establishment of blasticidin resistance; A ShSh ble gene from *Streptoalloteichus hindustanus* was used to replace the β-lactamase gene in pGFP::SW2, which both eliminated penicillin resistance and established zeocin resistance. Using blasticidin selection, we stably transformed plasmid-free serovar D with pGFPBSD/Z::SW2 in 3 independent trials. GFP-BSD fusion protein was detected in the transformants and the transformation restored the plasmid property to the plasmid-free serovar D. Thus, we obtained a shuttle vector with β-lactamase-free selection markers for transforming STI-causing serovars of *Chlamydia trachomatis*. The new shuttle vector and the successful transformation procedure provided in the current study will not only allow avoiding causing beta lactam resistance among wild type strains of C. trachomatis but will also provide important tools to investigate pathogenic mechanisms and develop vaccines directly using *C. trachomatis* serovars that are responsible for causing sexually transmitted chlamydial infections in humans. 

The BSD gene was cloned from the vector pLenti6.3_V5-TOPO [17] and fused to the downstream of the GFP gene in pGFPBSD/Z::SW2. As expected, GFP-BSD fusion protein was detected in the culture infected with the stable transformant CTD1-pGFPBSD/Z. The GFP-BSD fusion protein is known to be able to inactivate blasticidin, allowing selection for positive transformation with blasticidin [22]. Blasticidin, a nucleoside antibiotic produced by *Streptomyces griseochromogenes*, is a potent translational inhibitor in both prokaryotic and eukaryotic cells. Since most GFP-BSD fusion proteins were detected inside chlamydial inclusions (Figure 4A), a question arises as to how the GFP-BSD fusion protein conferred resistance of the infected host cells to the toxicity of blasticidin. Our hypothesis is that the intra-inclusion GFP-BSD fusion protein can adequately protect *C. trachomatis* from the blasticidin inhibition. Cells productively infected with chlamydia have been known to be highly resistant to apoptosis induction [21,26]. Thus, as a result of the continuing replication of chlamydia that produce GFP-BSD, the infected host cells became resistant to apoptosis induction by blasticidin, allowing the intracellular chlamydia to complete their growth cycle. However, the neighboring cells infected by chlamydia that do not produce GFP-BSD or cells which are uninfected should be highly sensitive to the toxicity of blasticidin. We noticed rounding up of these unwanted cells in a few hours after applying blasticidin. Another possibility is that there may be some leakage of GFP-BSD fusion protein from chlamydial inclusions into host cell. Although the amount is not detectable under fluorescence microscopy, trace amounts may be sufficient for protecting infected cells. It is worth noting that since blasticidin-mediated killing of host cells is independent of the intracellular chlamydial growth cycle, selection for drug-resistant clones with blasticidin is immediate and efficient. Indeed, targeting host cells with blasticidin has been used for selecting for viral recombinants [27]. Regardless of how exactly blasticidin selected for the GFP-BSD-producing chlamydia, the convenient transformation and selection procedures described in the current manuscript will be very useful for investigating the biology and pathogenesis of STI-causing serovars of *C. trachomatis*. 

The modification also includes a replacement of the β-lactamase gene in the pGFP::SW2 with a ShSh ble gene [23]. This replacement has led to both elimination of the original penicillin resistance and conferred zeocin resistance (ZeoR). Zeocin, a member of bleomycin family, causes cell death by intercalating into and cleaving DNA and it is effective in both bacterial and mammalian cells. We used zeocin to select for bacterial colonies that were transformed with the pGFPBSD/Z::SW2 plasmid since it has a very low inhibition concentration for bacteria. The incorporation of zeocin resistance makes the pGFPBSD/Z::SW2 shuttle vector versatile for future application. 

There are multiple applications for this new shuttle vector and transformation procedure. Since we have previously shown that the coding regions of Pgp3-5 can be replaced by multiple non-plasmid genes [11], one can use this shuttle vector to simultaneously introduce multiple vaccine antigens into a single chlamydial organism. For example, the major outer membrane protein genes from serovars E, F and I can be introduced into serovar D organisms. In this way, a single organism can express neutralization epitopes from multiple serovars. Similarly, this shuttle vector can be used to introduce microRNAs for selectively knocking down virulence factors. In all, these and other applications of the new shuttle vector will greatly facilitate the investigation of chlamydial pathogenesis and the development of vaccines.

## Supporting Information

Table 1 **Primers used in the current study.** Both forward and back primers for constructing pGFPBSD/Z::SW2 (A), screening GFP positive clones (B) and detecting the 8 plasmid genes & the chromosomal gene ct177 (C) were listed.
(XLS)
